# Personal Music Players Use and Other Noise Hazards among Children 11 to 12 Years Old

**DOI:** 10.3390/ijerph17186934

**Published:** 2020-09-22

**Authors:** Weronika Swierniak, Elzbieta Gos, Piotr Henryk Skarzynski, Natalia Czajka, Henryk Skarzynski

**Affiliations:** 1 Department of Teleaudiology and Screening, World Hearing Center, Institute of Physiology and Pathology of Hearing, 10 Mochnackiego Street, 02-042 Warsaw, Poland; w.swierniak@ifps.org.pl (W.S.); e.gos@ifps.org.pl (E.G.); n.czajka@ifps.org.pl (N.C.); 2 Heart Failure and Cardiac Rehabilitation Department, Faculty of Medicine, Medical University of Warsaw, 8 Kondratowicza Street, 03-242 Warsaw, Poland; 3 Institute of Sensory Organs, 1 Mokra Street, 05-830 Nadarzyn/Kajetany, Poland; 4 Department of Oto-Rhino-Laryngosurgery, World Hearing Center, Institute of Physiology and Pathology of Hearing, 10 Mochnackiego Street, 02-042 Warsaw, Poland; h.skarzynski@ifps.org.pl

**Keywords:** noise, noise induced hearing loss, personal music players, children

## Abstract

Exposure to loud music—due to widespread personal music players (PMPs) and noisy leisure activities—are major risk factors for noise induced hearing loss (NIHL) in adolescents. However, there is little evidence of the impact of noise on the hearing of younger children. This study aimed to explore an association between PMP use and hearing, and to identify other sources of noise among children. The study sample consisted of 1032 children aged 11–12 years old. Hearing thresholds were determined from 0.5 to 8 kHz. PMP use and other noise exposures were evaluated using a survey. We found that 82% of the children had a PMP, and 78% were exposed to noise when playing computer games. An audiometric notch was documented in 1.3% of the children. Only 11.5% of the children ever used hearing protection while engaged in noisy activities. We found no convincing evidence of an association between PMP use and hearing thresholds, although our results suggest that tinnitus may be an early sign of NIHL in young children. The study shows a need to provide children, their parents, and educators with knowledge of how to take care of hearing, including how to avoid and minimize noise exposure.

## 1. Introduction

Noise exposure is a common component of life which negatively affects human health. There are many studies that have described the effects of environmental noise and its association with health problems such as annoyance [1,2], sleep disturbance [3], hypertension and ischemic heart disease [4,5], and learning impairment in children [6,7]. Some people are exposed to constant but lower noise, not high enough to have a direct effect, but inducing various health problems [8,9].

Noise exposure is particularly detrimental to hearing. Noise-induced hearing loss (NIHL) can result from both a single sudden noise (acoustic trauma) or from long-term steady noise [10]. It can injure the eardrum or middle-ear ossicles, damage hair cells in the cochlea [11,12,13], and affect the auditory nerve and its myelin sheath [14]. NIHL is typically revealed by a notch in the audiogram at 4 kHz [15], which can spread to nearby frequencies at 3 and 6 kHz but with some hearing recovery at 8 kHz [16,17]. A genetic susceptibility to NIHL is being investigated [13,18], but most researchers focus their attention on environmental factors. For adults, occupational noise is the main consideration [19], but for children the impact of noisy leisure activities and use of personal music players (PMPs) is the primary aspect [20].

Many studies have shown that the prevalence of NIHL is considerable among young people. Data from a large-scale American study (1988–1994) indicated that 15.9% of children aged 12 to 19 years had hearing deficits attributable to noise exposure [21]. More recent data (2005–2006) from young Americans were quite similar, the rate being 16.8% [22]. In the Netherlands it has been found that 14.2% of a large cohort showed that children aged 9 to 11 years had hearing impairment possibly correlated with NIHL [23]. In Poland, 11.5% of 643 youths aged 13 to 18 years showed a notched hearing loss at 4 or 6 kHz, and it was significantly higher in those with heavy exposure to loud music (16.3%) compared to those with mild exposure (10.7%) [24].

Many children, adolescents, and young adults engage in noisy recreational activities (e.g., attending concerts, matches, discos, playing with noisy toys, and listening to loud music). As documented by Henderson et al. [22], exposure to loud noise or listening to music over headphones is an upward trend among young people—which is not surprising, given the widespread use of PMPs, such as MP3 players, iPods, and mobile phones. In 2016, le Clercq et al. [20] undertook a systematic review and meta-analysis of music-induced hearing loss and its symptoms in young people, although most of the 33 articles concerned adolescents and young adults. It was concluded that most studies reported no significant association between exposure to loud music and pure-tone air thresholds, although some studies showed a higher prevalence of tinnitus and decreased otoacoustic emissions in participants more exposed to music. Significantly poorer hearing thresholds were documented for PMP users than for non-users, and le Clercq et al. [20] finally conclude that the accumulated data suggest an association between music exposure and hearing loss in children.

Generally, there are few studies related to NIHL in younger children, and their conclusions are not strong [23,25,26]. There is no clear-cut evidence for association between recreational noise exposure and hearing loss in younger children. The aim of this study was to explore an association between personal music player use and hearing, and to identify other sources of noise among children.

## 2. Materials and Methods

### 2.1. Study Design

Since November 2007, the Institute of Physiology and Pathology of Hearing, commissioned by Warsaw City Hall, has conducted hearing screening in primary schools in Warsaw. The goal of the program is to increase the detection of hearing disorders, improve early diagnosis, and give equal opportunities to children by reducing or eliminating the adverse consequences of hearing impairment [27,28]. The main objective of the hearing screening program is to assess the hearing of the studied population. For the purposes of the present article we retrospectively analyzed a portion of the data obtained in one year of the program to assess the noise hazard among sixth-grade children who were due to complete their primary education.

Prior to testing, the children’s parents were informed of the testing procedure and were asked to give written consent for their children to participate in the hearing examinations. The study was conducted following the Declaration of Helsinki and was approved by the Research Ethics Committee (KB.IFPS:28/4/2018).

### 2.2. Audiometric Testing

**Figure 1:**
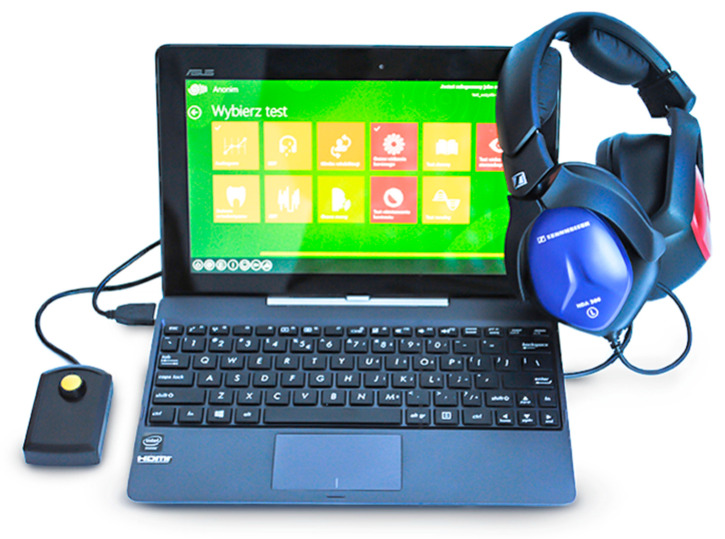
Platform for Sensory Organs Examination.

Screening pure tone audiometry was conducted in a quiet room allocated by the school headmaster. The examination was performed on the Platform for Sensory Organs Examination [27,29,30] connected to the “SZOK”^®^ central database [31]. The system is based on a powerful central computer with multiple portable computers communicating with it via the Internet. Each portable device is equipped with software that allows it to perform pure-tone audiometry. The platform includes a response button and is set up with Sennheiser HDA300 audiometric headphones which provide effective acoustic isolation from background noise. The platform is shown in Figure 1.

For each ear separately, air conduction thresholds at conventional octave frequencies from 0.5 to 8 kHz were determined with the same paradigms as in a previous study [27,29,32] as well as at half-octave frequencies of 3 and 6 kHz. Measurements at 125 and 250 Hz were omitted as being less useful and vulnerable to noise disturbance [33].

#### Presence of a Notched Audiogram

Children were studied for the presence of a high-frequency notch. According to Niskar et al. [21], a notch was considered to be present when three conditions were met: (i) thresholds at 0.5 and 1 kHz were lower (better) than 15 dB; (ii) thresholds at 3, 4, or 6 kHz were 15 dB or higher (poorer) than the poorest threshold at 0.5 or 1 kHz; and (iii) the threshold at 8 kHz was at least 10 dB lower (better) than the poorest threshold at 3, 4, or 6 kHz.

### 2.3. Survey

A brief survey used was developed by an ENT specialist and audiologists working in Institute of Physiology and Pathology of Hearing in Poland. Developing it we based on our experience on screening examination among school children which we have been conducted for many years. The survey contained 8 questions (6 closed-ended and 2 open-ended questions) concerned PMP use, exposure to other sources of noise, tinnitus experience, and use of hearing protection.

The survey was completed by the children before audiometric testing. PMP use was assessed on the basis of 6 items reported by the participants: 1. Do you use PMP? (yes, no). 2. Which kind of headphones do you use the most frequently? (headphones, earphones). 3. Mark on the line the volume level you normally set on your PMP (the mark was then converted into percent). 4. How often do you listen to music through PMP on a specified level? (every day, 4–6 times a week, 2–3 times a week, once a week, less than once a week, I do not to listen music). 5. How many hours a day do you listen to music through the PMP? (above 6 h, 5–6 h, 3–4 h, 1–2 h, less than 1 h). 6. What are your activities when you use your PMP?

Exposure to other sources of noise was evaluated by questioning how often the children participated in various noisy activities (listed in Table 1). A question about tinnitus was: Do you hear tinnitus, whistles, squeaks or other sounds when in quiet? (never, rarely, sometimes, often, always). The last question concerned use of hearing protection in noisy environments, e.g., concerts, matches, disco, shooting range (never, rarely, sometimes, often, always, not applicable).

### 2.4. Participants

There were 1032 children (546 girls and 486 boys), made up of 265 children (26%) aged 11 years and 767 (74%) aged 12 years.

### 2.5. Statistical Analysis

Hearing thresholds between PMP users and non-users were compared using a *t*-test for independent samples. A chi-square test for independence was made to determine if there was a significant association between tinnitus and PMP use. Statistical significance was set at a *p*-value of 0.05. Analysis was conducted using IBM SPSS Statistics v. 24 (IBM Corp., Armonk, NY, USA).

## 3. Results

### 3.1. Personal Music Player Use

Of 1032 participants, 82% (*n* = 849) had a PMP, while 183 children (18%) did not. Of children having a PMP, 48% (*n* = 411) said they used headphones, while 52% (*n* = 438) used earphones.

The participants estimated the volume level normally set by them on their PMP as 50.8% on average (SD = 21.2, median 45.3%). The frequency of PMP use was as follows: 86 children (10.1%) reported they listened to music every day; 58 (6.8%) 4–6 times a week; 187 (22%) 2–3 times a week; 119 (14%) once a week; 126 (14.8%) less than once a week; and 273 (32.2%) reported they did not listen to music. The PMP listening time was: 3 children (0.3%) reported they listened to music 5 or more hours a day; 23 (2.7%) 3–4 h a day, 101 (11.9%) 1–2 h a day; and 449 (52.9%) less than 1 h a day.

The most common situations when using a PMP were: Traveling by car (31.6%, *n* = 268) or public transport (29.3%, *n* = 249); playing computer games (27.1%, *n* = 230); playing on a phone (19.7%, *n* = 167); walking (19.1%, *n* = 162); doing homework (13%, *n* = 110); playing sport (11.5%, *n* = 98); sleeping (9.7%, *n* = 82); and reading (3.9%, *n* = 33).

Comparisons of hearing thresholds across all tested frequencies for PMP users and non-users are shown in Table 2.

Hearing thresholds in the PMP users and non-users were generally similar, except at 3 kHz in the left ear and 4 and 6 kHz in the right ear. Poorer average hearing thresholds at above mentioned frequencies were observed in the non-user group, however the differences were less than 2 dB.

### 3.2. Tinnitus

There was a significant difference between the PMP users and non-users in terms of experiencing tinnitus: *χ*^2^(4) = 16.87; *p* = 0.002. Some 76% of PMP non-users never experienced tinnitus, but among the PMP users the rate was lower (61.6%). At the same time, the rate of experiencing tinnitus often or always was higher in PMP users (3.2%) in comparison to PMP non-users (1%). The data are shown in Table 3.

### 3.3. Other Sources of Leisure Noise

Table 1 shows how often the children undertook or participated in various noisy leisure activities. As can be seen, the most common source of leisure noise, other than PMP use, was playing computer games. Attending musical and sporting events was much rarer, and other activities were very rare.

### 3.4. Hearing Protection Use

Of 1032 participants, 701 (67.9%) reported they never used hearing protection in noisy environments (e.g., concerts, matches, disco, and shooting range); 59 (5.7%) used them rarely; 45 (4.4%) sometimes; 9 (0.9%) often; 6 (0.6%) always; and 212 (20.5%) not applicable. That means that only 119 of the 1032 children (11.5%) ever used any hearing protection.

### 3.5. Audiometric Notches

Audiometric notches were observed in 13 (1.3%) of the children (7 in the left ear, 2 in the right ear, and 4 bilaterally). Notched audiograms were found in 6 girls and 7 boys. Of the 13, 8 used a PMP and 5 did not; 4 wore headphones and 4 wore earphones. Generally, we did not observe any specific noisy leisure time activity among the 13 children with audiometric notches. As in the whole study group, the most common noisy activity was playing computer games. The 13 children with audiometric notches did not stand out from the other children.

## 4. Discussion

The aim of the present study was to determine the impact of PMP use on children’s hearing and to identify other problematic sources of noise. We used data collected during hearing screening of children aged 11–12 years old who were completing the last grade of primary school in Warsaw. We found that 82% of the children had a PMP, and 78% of the children were exposed to noise when playing computer games. We therefore assume that exposure to noise is a common problem among children.

In 2016 le Clercq et al. [20] undertook a systematic review and meta-analysis of music-induced hearing loss and its symptoms in children, adolescents, and young adults. The authors examined 33 articles which met strict inclusion criteria and had sufficient methodological quality. They reported a prevalence of music-induced hearing loss of 0% to 12.6% (when evaluating average thresholds) and 14.2% to 34.9% when considering an increase in threshold at one frequency or more. The weighted average of the prevalence of music-induced hearing loss was 9.6% and for high frequency hearing loss 9.3%. The authors concluded that on the one hand, most studies reported no significant association between exposure to loud music and pure-tone air thresholds, but on the other hand they showed in the meta-analysis that PMP users had significantly poorer hearing thresholds than non-users at high frequencies (4, 6, 8, 10, 12.5, and 16 kHz).

Our findings do not confirm the harmful effect of PMP use on hearing. Pure tone hearing thresholds were not significantly elevated in PMP users in comparison to PMP non-users. In both groups hearing thresholds were generally similar, however slightly poorer hearing (about 2 dB) at three frequencies was observed in children who did not use a PMP. We must admit we did not expect such a result, even though no consensus exists regarding the risk associated with personal listening devices in causing NIHL. Mostafapour et al. [34] state that the majority of young PMP users are at low risk of NIHL, but point out that NIHL is an additive process and even slight effects of continued exposure to noise may accumulate over many years. Le Clercq et al. [23] also showed no association between PMP use and notched audiograms in a large cohort of children aged 9–11 years, but they did find an association between PMP use and high-frequency hearing loss (average threshold at 3, 4, 6, and 8 kHz). Similarly inconclusive are the results of a study conducted by Båsjö et al. [25] concerning listening habits in 415 nine-year-old children. The study showed that hearing thresholds for children who often listen to music with headphones were significantly poorer in comparison with children who did not listen with headphones, but only in the right ear.

Cone et al.’s study [26] which examined 6581 children aged 7–11 years showed that exposure to recreational noise (e.g., noisy toys, firecrackers, referee whistles, lawn mowers, and power tools) was slightly higher in children with mild sensorineural hearing loss (18%) than in children with normal hearing (10.2%). Use of personal stereos was also a significant risk factor for mild sensorineural hearing loss with an odds ratio of 1.7 (although it should be noted that the 95% confidence interval was from 1.0 to 3.0). In summary, the results are equivocal.

Our children were 11–12 years old. We think they had not been using a PMP for a long time and had not yet developed a habit of listening for long periods. Over half of the participants listened to music through their PMP for less than 1 h a day, and only 10% listened to music every day. They did not seem to have used their PMP long enough to impair their hearing thresholds. Furthermore, PMP users reported that the volume level they used was on average about 50%, so the noise level was probably insufficient to cause damage to hearing over a short time.

A quite similar view was presented by You et al. [35] after a study of 1009 Korean college students who used personal listening devices. Harrison [36] supposed that, in the short term, the impact of noise exposure may not be apparent and not manifest immediately, but the accumulated effects may eventually lead to serious hearing deficits later on. This opinion sounds plausible.

Our study showed that PMP users more often experienced tinnitus than PMP non-users. The link between tinnitus and noise has been documented by many researchers. Mazurek et al. [37] revealed that 83% of adult tinnitus patients had high-frequency hearing loss corresponding to the pattern found with NIHL. Similar evidence has been given by other researchers [38,39,40,41] but all these studies were done in adult subjects. Knowledge of noise-induced tinnitus in children remains limited to only a few studies [42]. Holgers et al. [43], Juul et al. [44], and Nemholt et al. [45] investigated noise-induced tinnitus in children, but only after listening to loud music or other loud sounds, so their results refer to temporary threshold shift (TTS) rather than to NIHL. More interesting are the findings of Coelho et al. [46] who investigated children aged 5 to 12 years old. They conclude that a history of noise exposure was a risk factor for tinnitus (an odds ratio of 1.8) and for troublesome tinnitus (an odds ratio of 2.8); however, it was unclear how a history of noise exposure was defined. Our findings suggest that tinnitus may precede full-blown NIHL and be an early sign of hearing impairment following later. This hypothesis is worth verifying, preferably in a longitudinal study.

In our study we asked children about several activities described in the literature as being noisy and a risk factor for NIHL. The main finding was that children often play computer games—78% engaged in this activity at various levels. Among the harmful effects that computer games may have on children’s health, research has pointed to aggressive behavior and thoughts, emotional problems, hyperactivity, and inattention [47,48]. The potential effect of playing computer games on hearing has not been widely discussed, but Iannace et al. [49] showed that game users are highly exposed to noise and potential damage to hearing depends on a game’s sound intensity and exposure time. Our study revealed that other leisure activities (e.g., attending music or sporting events) were not frequently undertaken by the children, which seems understandable taking into account the young age of our cohort.

We found that only 11.5% of the children ever used hearing protection while engaged in noisy activities. Bogoch et al. [50] found that only about 20% of attendees of rock concerts ever wore hearing protection at such events. Similar data was given by Olsen–Widen et al. [51] who found that about 30% of adolescents used hearing protection at concerts. We think that, for younger children, the parents’ attitude and health education play key roles. Nowadays, children routinely use bike helmets, seat belts, or car seats to prevent possible injury, and we think that hearing protection devices should also be promoted. Teachers and parents need to teach children health-oriented behaviors of how to avoid and minimize the harmful effects of noise.

We found notched audiograms in only 1.3% of the children. This rate is lower than in other studies—4.5% by le Clercq et al. [23] in a study sample of 3116 children 9–11 years old and the 16.8% figure found by Henderson et al. [22] in a sample of 4310 adolescents 12–19 years old. However, Twardella et al. [52] found that only 2.3% of 2143 adolescents displayed a notched audiogram. This inconsistency, especially with the le Clercq et al. [23] results obtained in younger children, shows that there is a need to accumulate evidence about noise exposure and its impact on children’s hearing from various countries.

Our study has certain limitations. We gathered data during a hearing screening conducted in primary schools in Warsaw, the capital of Poland. The aim of the screening was to detect possible hearing impairment and refer pupils with positive results for further diagnostic testing. Audiological testing consisted only of pure tone audiometry, which is considered the gold standard for evaluating hearing levels [53,54], but otoacoustic emissions might be a more sensitive way of detecting hearing impairment related to noise exposure. Although our study sample was rather large, it is not representative of the whole population of Polish children aged 11–12 years; it only comprised children living in the biggest Polish city. Another limitation is a reliance on self-reporting by the children of their exposure to noise. Our study was observational, not an experiment, and we were not able to precisely define the frequency, duration, and loudness of exposure to loud music and other sources of noise. Some scalar quantity of noise would be beneficial to the study. Despite these drawbacks, this study is noteworthy because the evidence of the effects of noise exposure in younger children is currently scarce. Accumulating a range of results from different settings will help create guidelines of how to determine NIHL in children and how to prevent it.

## 5. Conclusions

Our findings show that children aged 11–12 years are exposed to leisure noise and PMP use is its considerable source. However, only 10% of the children reported using PMP every day, and 53% of the PMP users listened to music less than hour a day. The volume level normally set by the children was on average 50.8%. We did not find a convincing association between pure-tone hearing thresholds and PMP use, and notched audiograms were documented in only 1.3% of the children. However, the results suggest that tinnitus, which was slightly more frequent in PMP users, might be an early sign of NIHL. The most common source of leisure noise, other than PMP use, was playing computer games (78% of the children). Only 11.5% of the children ever used hearing protection while engaged in noisy activities. There is a need for future research to objectively quantify noise exposure in children of this age. Particular attention should be paid to prevention of NIHL among young children.

## Figures and Tables

**Table 1:** Frequency of noisy leisure activities.

Activity	Every Day	4–5 Times a Week	2–3 Times a Week	Once a Week	Once a Month	Several Times a Year	No; Not Applicable
Practising musical instruments	10 (1.0)	3 (0.3)	10 (1.0)	20 (1.9)	6 (0.6)	21 (2.0)	962 (93.2)
Hunting, shooting	-	-	3 (0.3)	5 (0.5)	8 (0.8)	30 (2.9)	986 (95.5)
Noisy power tools (e.g., chain saw, electric drill)	1 (0.1)	1 (0.1)	2 (0.2)	9 (0.9)	10 (1.0)	49 (4.7)	960 (93.0)
Concerts, music events	-	-	1 (0.1)	2 (0.2)	24 (2.3)	258 (25.0)	747 (72.4)
Computer games (Playstation, X-Box, Nintendo)	116 (11.2)	114 (11.0)	246 (23.8)	156 (15.1)	101 (9.2)	74 (7.2)	225 (21.8)
Using caps, fireworks, firecrackers	-	-	4 (0.4)	1 (0.1)	8 (0.8)	262 (25.4)	757 (73.4)
Grass-cutting with lawnmower	-	-	-	14 (1.4)	53 (5.1)	132 (12.8)	833 (80.7)
Playing slot machines	-	-	2 (0.2)	2 (0.2)	12 (1.2)	55 (5.3)	961 (93.1)
Matches, sporting events	4 (0.4)	3 (0.3)	22 (2.1)	52 (5.0)	96 (9.3)	237 (23.0)	618 (59.9)
Disco	-	-	-	5 (0.5)	41 (4.0)	554 (53.7)	432 (41.9)

Children playing computer games were divided into two groups: Those who played 2–3 times a week or more often (*n* = 476), and those who played at most once a week or not at all (*n* = 556). Their hearing thresholds did not differ significantly.

**Table 2:** Hearing thresholds (dB HL) in personal music player (PMP) users and non-users.

		PMP Users (*n* = 849)		PMP Non-Users (*n* = 183)		*t*	*p*
	kHz	M	SD	M	SD		
LE	0.5	11.18	5.55	11.72	6.01	1.17	0.242
	1	8.43	5.75	9.07	6.46	1.33	0.184
	2	7.39	6.62	7.68	6.87	0.53	0.598
	3	7.72	7.16	9.04	8.24	2.21	0.028
	4	6.02	6.88	7.16	8.09	1.96	0.051
	6	7.70	7.16	8.61	8.08	1.52	0.128
	8	9.13	8.27	9.81	8.95	0.99	0.320
RE	0.5	11.31	5.08	11.17	5.43	0.33	0.741
	1	9.09	5.39	9.26	5.71	0.39	0.693
	2	7.20	5.99	7.73	5.95	1.10	0.272
	3	6.90	6.27	7.57	5.79	1.33	0.183
	4	5.23	6.36	6.39	7.82	2.15	0.032
	6	7.00	6.32	8.61	9.02	2.87	0.004
	8	8.23	7.91	8.69	9.07	0.69	0.492

LE, left ear; RE, right ear; M, mean; and SD, standard deviation.

**Table 3:** Prevalence of tinnitus in PMP users and non-users.

	Never	Rarely	Sometimes	Often	Always
PMP users (*n* = 849)	523 (61.6)	168 (19.8)	131 (15.4)	20 (2.4)	7 (0.8)
PMP non-users (*n* = 189)	139 (76.0)	30 (16.4)	12 (6.6)	1 (0.5)	1 (0.5)
